# Multidisciplinary management of a large pheochromocytoma presenting with cardiogenic shock: a case report

**DOI:** 10.1186/s12894-019-0554-5

**Published:** 2019-11-20

**Authors:** Umberto Maestroni, Francesco Ziglioli, Marco Baciarello, Valentina Bellini, Raffaele Dalla Valle, Simona Cataldo, Giada Maspero, Elena Bignami

**Affiliations:** 1 Department. of General and Specialty Surgery, Urology Unit, University Hospital of Parma, Parma, Italy; 2 Department of Medicine and Surgery, Anesthesia, Critical Care and Pain Medicine, University of Parma, Parma, Italy; 3 Department of Medicine and Surgery, Hepatobiliary Surgery Unit, University of Parma, Parma, Italy; 4 Department of Medicine, Endocrinology and Metabolic Diseases Unit, University Hospital of Parma, Parma, Italy

**Keywords:** Pheochromocytoma, Adrenalectomy, Cardiogenic shock, Case report

## Abstract

**Background:**

Pheochromocytoma is well-known for sudden initial presentations, particularly in younger patients. Hemodynamic instability may cause serious complications and delay a patient’s ability to undergo surgical resection. Larger tumors present a further challenge because of the risk of catecholamine release during manipulations. In the case we present, increases in systemic vascular resistance caused cardiogenic shock, and the size of the lesion prompted surgeons to veer off from their usual approach.

**Case presentation:**

A 38-year-old female patient was admitted to our intensive care unit with hypertension and later cardiogenic shock. Profound systolic dysfunction (left ventricular ejection fraction of 0.12) was noted together with severely increased systemic vascular resistance, and gradually responded to vasodilator infusion. A left-sided 11-cm adrenal mass was found with computed tomography and confirmed a pheochromocytoma with a meta-iodo-benzyl-guanidine scintigraphy. Surgical treatment was carefully planned by the endocrinologist, anesthesiologist and surgeon, and was ultimately successful. After prolonged hemodynamic stabilization, open adrenalectomy and nephrectomy were deemed safer because of lesion size and the apparent invasion of the kidney. Surgery was successful and the patient was discharged home 5 days after surgery. She is free from disease at almost 2 years from the initial event.

**Conclusions:**

Large, invasive pheochromocytoma can be safely and effectively managed with open resection in experienced hands, provided all efforts are made to achieve hemodynamic stabilization and to minimize. Catecholamine release before and during surgery.

## Background

The classical triad of headache, sweating and hypertension in patients with cathecolamine-secreting adrenal tumors is hardly ever seen lately. In an era of aggressive pharmacological management of primary hypertension, only half pheochromocytoma patients are diagnosed during workup of blood pressure anomalies [1] Occasionally, however, patients present with extremely elevated blood pressure and heart rate, causing single or multiple organ failure in what has been termed “pheochromocytoma crisis” [2].

While surgical resection of large, symptomatic adrenal masses is standard practice, the choice of a laparoscopic vs open surgical approach is still controversial [3, 4].

We present the case of a patient whose first symptoms were catastrophic hypertension and tachycardia leading to cardiogenic shock. We will discuss our decision to proceed with open resection and the importance of a MDT in this context. This manuscript is written in accordance to the 2018 SCARE statement [5].

## Case presentation

A 38-year-old woman of Eastern European descent was admitted to the emergency department of a peripheral hospital for headache, dizziness, nausea and vomiting. She had a history of migraine and she had taken NSAIDs in the previous days to mitigate the headache. She was a secundipara with two vaginal deliveries, and had no further medical history.

On presentation she was hypertensive (170 over 130 mmHg) and tachycardic (130 bpm); peripheral blood oxygen saturation was low for her age at 90% in room air, and her tympanic temperature was 36.7 °C. She was rapidly approaching respiratory fatigue, with generalized cyanosis, accessory muscle recruitment and orthopnea. Peripheral pulses were noted to be barely palpable bilaterally. On echocardiography, global systolic function was depressed, with an ejection fraction of 0.25. Electrocardiogram showed mild ST-segment elevation in V5 and V6. The patient was initially hypervigilant and anxious, but her mental status deteriorated to the point that she was tracheally intubated to secure airway patency; there were no focal neurological deficits.

She was transferred to the ICU of the tertiary medical center, the University Hospital of Parma.

The main finding during secondary examination was a severely impaired systolic function (ejection fraction 0.12, calculated using a modified Simpson’s rule). As systolic BP reached values above 220 mmHg, sodium nitroprusside and labetalol were started in continuous infusion to lower BP and HR, respectively, and a pulmonary artery catheter was inserted. Thermodilution confirmed the diagnosis of cardiogenic shock due to severely increased peripheral vascular resistance.

**Fig. 1:**
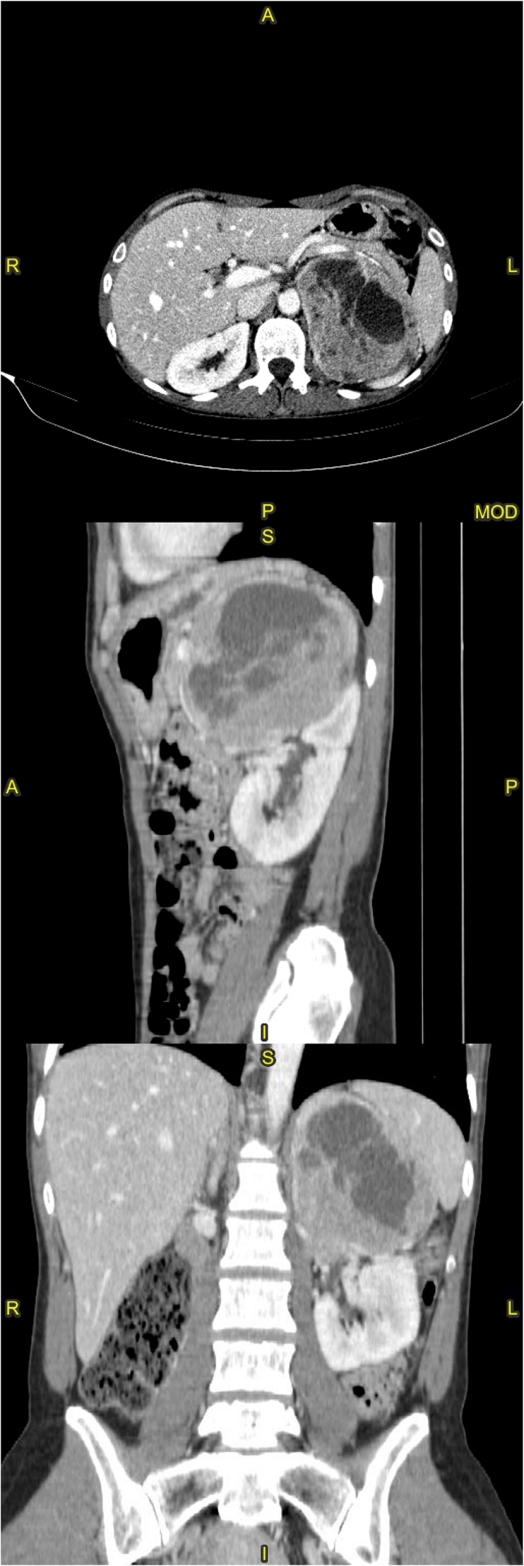
Multiplanar reconstruction of a contrast-enhanced abdominal computed tomography scan. The left adrenal mass has a maximum diameter of 11.2 cm, and is seen displacing the gastric fundus antero-superiorly and the spleen laterally; cleavage from the kidney is unclear around the pelvis in the coronal image, suggesting cancerous infiltration

Because of the patient’s unremarkable medical history and abruptness of the crisis, a total body CT scan was requested in search of a diagnosis. The brain scan was negative; lungs were remarkable for diffuse ground glass infiltrates; finally, a 11.4-cm adrenal mass occupying the superior cap of the left kidney was found. It had cystic and solid components (Fig. 1). There were also areas of increased bone density in the the inferior endplates of the D12, L1 and L3 vertebrae.

**Fig. 2:**
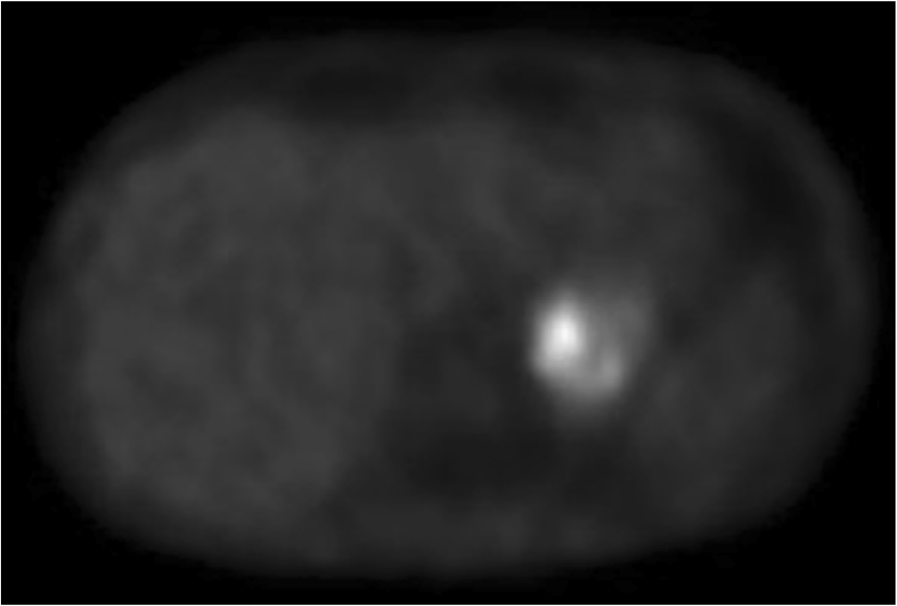
[123I]-MIBG gamma ray emission scan showing intense emission from a left-sided adrenal pheochromocytoma extending into the ipsilateral kidney

Urine analysis confirmed the hypothesis that norepinephrine levels were particularly elevated with respect to epinephrine. The patient then underwent a [123I]-MIBG to formalize the diagnosis of left-sided adrenal pheochromocytoma (Fig. 2) extending to the ipsilateral kidney.

The patient was extubated on the 4th day of admission, but she required noninvasive CPAP ventilation to maintain adequate oxygenation and avert respiratory muscle fatigue, despite her left ventricular ejection fraction having increased to 0.42. Her ICU stay was complicated by right lower lobe VAP which prolonged weaning from ventilation and delayed surgery, in order to complete a full course of vancomycin and piperacillin/tazobactam. Intravenous vasoactive drugs were weaned over 2 weeks and substituted with doxazosin 4 mg *tid*, bisoprolol 5 mg and amlodipine 10 mg daily. Blood pressure was stabilized but persistently elevated, around 150/95 mmHg, with HR closer to the lower normal range.

Since [123I]-MIBG scans have inferior sensitivity for pheochromocytoma metastases [6] and the disease was high-grade, a confirmatory [18F]-DOPA PET scan was also performed as soon as the patient was extubated. Both studies showed no uptake by the vertebral lesions, whereas the primary tumor had consistently increased signal.

Once pneumonia resolved and hemodynamics were confirmed stable, some 20 days after admission, the patient was transferred to an internal medicine ward and a MDT was assembled to take charge of the patient. A radiologist, a urologist, an endocrinologist and an anesthesiologist convened to offer open resection of the malignancy, to include nephrectomy because of the apparent invasion, under general anesthesia with epidural analgesia. It was convened that the combined results of the functional and morphological imaging studies did not warrant a vertebral biopsy, particularly in such a high-risk patient, and active monitoring was proposed. Our patient then consented to surgery with planned postoperative surgical ICU admission.

In the preoperative holding area, an epidural catheter was sited at the T10–T11 interspace and loaded with 10 μg of sufentanil, a potent short-acting opioid; the anesthesiologist opted to reserve LA administration until hypertensive crises developed. Monitoring included radial and pulmonary artery catheters, the latter to better assess systemic vascular resistances and systemic oxygen delivery vs consumption in case of hypotension after tumor resection.

As expected, a hypertensive crisis developed during tumor isolation, and it was managed with continuous infusions of sodium nitroprusside and esmolol, as well as generous lidocaine boluses (up to 80 mg every 10–15 min) via the epidural catheter, to induce pharmacological sympathectomy with vasodilation in the lower half of the body. During such management, cardiac systolic function remained at the lower end of the normal range, as did mixed venous saturation, which was never below 70%.

A left subcostal incision was chosen, which provided the surgeons with optimal exposure, confirming its usefulness in the excision of larger adrenal masses. Left kidney invasion was confirmed upon direct inspection. The renal vein was ligated first in order to block incretion of catecholamines, followed by the artery. The tumor’s size and the inflammatory reaction around it complicated cleavage, which took more time than expected; resection *en bloc* with the kidney was nevertheless completed in 100 min, with an estimated blood loss of 550 ml.

Hydrocortisone (200 mg daily in continuous infusion after an intraoperative 100-mg loading dose) was selected to transition the patient during the expected transient acute adrenal insufficiency, in the first 48 h. Hypotension developed as soon as the renal vein was ligated, and required norepinephrine infusion in addition to discontinuation of the previous short-acting vasoactive drugs. One additional 100-mg hydrocortisone bolus was also given during surgery.

The patient was transferred to the surgical ICU for monitoring. She was weaned from ventilation and extubated shortly after admission. Norepinephrine was also gradually discontinued within the first 18 h. Doxazosin and amlodipine were withheld the same day, whereas bisoprolol was tapered off over 3 days. Hydrocortisone was tapered to 100 mg/day and replaced with same-dose oral cortisone; total cortisol and ACTH levels were monitored to guide therapy and maintained within normal ranges. On postoperative day 1, slightly elevated serum troponin I and urine dopamine concentrations were detected; in light of the overall satisfactory clinical condition, this was attributed to the mineralocorticoid activity of the exogenous steroids. On postoperative day 2 the patient was transferred to the Urology floor. Her stay there was unremarkable, and she was discharged home in good conditions 4 days later. One month later, urinary metanephrine and particularly normetanephrine levels were half the preoperative values, yet still elevated (~ 900 μg/24 h), but there were no associated signs or symptoms; cortical hormones were within the normal range throughout the whole postoperative period.

Histopathology findings were adrenal pheochromocytoma and chronic pyelonephritis, which had resembled malignant invasion in imaging studies and during surgery. Immunohystochemistry analyses were positive for chromogranin A, synaptophysin and neuron-specific enolase. Final PASS [7] was 10. The score is compatible with high risk of malignant behavior. In the absence of obvious metastases and of recurrent signs or symptoms, the MDT opted to offer close follow-up and to withhold further treatment.

Two years after diagnosis and surgery, the patient has discontinued all drugs and enjoys a disease-free life. Metabolic tests were normalized since the first follow-up, 6 months after surgery. She was tested for genetic variants of lactate dehydrogenase, succinate dehydrogenase and for mutations associated with multiple endocrine neoplasia, type 2; results were negative. She is being followed up with urine cathecolamine tests twice a year, and surveillance magnetic resonance imaging every other year.

## Discussion and conclusions

Pheochromocytoma is a rare neuroendocrine tumor, which may occur sporadically [8] but is more typically associated with inheritable syndromes. A significant proportion of pheochromocytomas may be clinically silent, with up to 58% classifying as adrenal “incidentalomas” [9]. While hypertension is a typical feature of symptomatic pheochromocytoma, its unexpected onset in certain patients may be mistaken for more typical conditions for that population; migraine was the first diagnosis in our patient, and in the absence of a trigger event, physicians could not immediately associate her hypertensive crisis and subsequent shock to the presence of cathecolamine-producing tumor. Abdominal surgery is likely the most common trigger, but there are reports associating crises with glucocorticoid administration [10], anesthesia induction [11] and even low back massage [12]. Our patient presented with life-threatening signs and symptoms, and the successful management was first and foremost the result of coordination between specialties. One of the peculiar aspects of this case is how the patient stayed in the ICU well after the initial crisis resolved. This was initially a deliberate choice which allowed stable hemodynamics control and optimization of the patient’s respiratory function; development of VAP caused an unexpected delay in discharge and surgery, but its management in the ICU (where advanced monitoring and treatment are possible) averted any trigger events it might have caused.

Another crucial point in management was the choice of the surgical approach. This was not the sole decision of a surgeon, but instead it was the result of contributions from all members of the MDT. Since the original descriptions by Garner and coll [13]. VL adrenalectomy has gradually become the standard of care for resections of large adrenal masses [14–16]. Advantages of laparoscopy include shorter length of hospital stay, lower morbidity and postoperative pain, and better cosmetic results. In the case of clinically active pheochromocytoma, however, greater caution is warranted, as it is a hormone-secreting tumor in which size can make a significant difference when choosing the surgical approach [17]. It is generally agreed that a maximum pheochromocytoma diameter > 6 cm identifies a “large” tumor, and that VL resection is feasible, though technically challenging [18–20]. Conzo et al. [21], as well as de Fourmestraux and coll [18], retrospectively compared results of VL resection in large vs normal (*ie*, < 6 cm) pheochromocytomas; they reported no significant differences in terms of blood loss or surgical time, which is also, in this context, a (poor) surrogate measure of the extent of tumor manipulation. Other more recent studies demonstrate significantly longer surgery duration and more blood loss with laparoscopy [3, 4]. Perhaps counterintuitively, laparoscopic resection does not invariably result in greater hemodynamic stability [22, 23]. This may be surprising for intraoperative hypertension/tachycardia (often the product of tumor manipulations), as one would expect laparoscopic surgery to be “gentler” on the mass. However, hypotension should be expected to be as likely in VL as in open surgery, since it is initially associated with renal vein ligature and interruption of catecholamine incretion, followed by relative adrenal insufficiency later on.

Our patient’s tumor had a maximum diameter of 11.4 cm, very large by common criteria, and it appeared to infiltrate the kidney diffusely. Although the surgeon, UM, has good experience with laparoscopic adrenalectomy [15, 16], the open approach allowed us to minimize tumor mobilization until the renal vein was ligated, to a degree that we believe would be unlikely in laparoscopic resection of such a large mass. Minimization of catecholamine discharge during surgery was felt to be the top safety concern for this patient, seeing as how it had caused vasoconstriction to the point of cardiogenic shock even in the absence of a trigger event.

Finally, the catastrophic presentation of our patient’s pheochromocytoma is unusual in that her cardiogenic shock did not fit the typical picture of adrenergic or stress-related cardiomyopathy (also known as TS). In an analysis of 80 case reports of pheochromocytoma-associated TS, 20% demonstrated globally reduced systolic function [24]; however, in the International Takotsubo Registry, out of 1750 patients, none were found to have global systolic depression, as opposed to focal ballooning [25].

We hypothesize that in a sizable proportion of pheochromocytoma crisis patients, acute heart failure results from severely increased systemic vascular resistance, as was the case with our patient. Takotsubo in pheochromocytoma is typically focal and may be a result of myocardial inflammation due to high levels of circulating catecholamines [26]. By contrast, our patient responded well to vasodilators and she did not require the use of inotropic agents. Her left ventricular contractility recovered rapidly as BP was stabilized, whereas many patients with pheochromocytoma-related TS suffer from recurrences (17.7% in Y-Hassan’s review) [24]. A combination of alpha-adrenergic blockade as well as non-antiadrenergic vasodilators are recommended in addition beta-blockers to minimize the risk of further hemodynamic deterioration [27]; to this end, an older drug such as labetalol is still a good choice.

Our choice of monitoring the vertebral lesions is highly debatable; since the primary tumor had an elevated PASS score, a biopsy might have been the most prudent option. However, the lesions showed no uptake of markers which were avidly absorbed by the primary lesion, and there weren’t any osteolytic regions within the sclerotic areas [28]. Bone sclerosis is a relatively common sign found in the general population, especially in the presence of degenerative disc disease, whether or not they suffer from low back pain [29]. Had the vertebral lesions turned out to be metastases, there is anecdotal evidence of symptom improvement at 6–12 months, but no clear survival benefit [30]. In our case, complete resection would have entailed a three-level corpectomy, which we doubt would have been feasible, or at least compatible with acceptable quality of life.

In conclusion, we report a case of pheochromocytoma initially presenting with headache and hypertension, rapidly evolving into cardiogenic shock secondary to increased afterload. Blood pressure control with alpha- and beta-adrenergic antagonists led to stabilization of myocardial function but the patient’s course was complicated by VAP. A MDT selected what they felt was the safest approach to surgery given the large mass and its secretory potential; open adrenalectomy and nephrectomy were performed with alpha- and beta-blockers, epidural analgesia and general anesthesia with advanced cardiovascular monitoring.

Aside from the specific case presentation, the concurrent involvement of different specialists was instrumental for successful management. Preoperative stabilization was obtained by intensivists and endocrinologists; the anesthesia team treated intraoperative hemodynamic imbalances which might have resulted in serious complications; the surgical team’s judgment and experience helped make the choice of the technique, which resulted in minimal impact on the patient. Our case shows that multidisciplinary management is critical for minimizing the risk of complications in decompensated pheochromocytoma.

## Data Availability

Not applicable.

## Abbreviations

ACTH: adrenocorticotropic hormone
BP: blood pressure
CPAP: continuous positive airway pressure
DOPA: 3,4-dihydroxyphenylalanine
HR: heart rate
ICU: intensive care unit
LA: local anesthetic
MDT: multidisciplinary team
MIBG: meta-iodo-benzyl-guanidine
OR: operating room
PASS: Pheochromocytoma of the Adrenal gland Scaled Score
PET: positron-emission tomography
TS: takotsubo syndrome
VAP: Ventilator-associated pneumonia
VL: video laparoscopy
